# Apoptotic Effects of Antilymphocyte Globulins on Human Pro-inflammatory CD4^+^CD28^−^ T-cells

**DOI:** 10.1371/journal.pone.0033939

**Published:** 2012-03-30

**Authors:** Christina Duftner, Christian Dejaco, Paul Hengster, Klaudija Bijuklic, Michael Joannidis, Raimund Margreiter, Michael Schirmer

**Affiliations:** 1 Department of Internal Medicine, Clinic of Internal Medicine I, Innsbruck Medical University, Innsbruck, Austria; 2 Department of Internal Medicine, General Hospital of Kufstein, Kufstein, Austria; 3 Department of Rheumatology, Medical University Graz, Graz, Austria; 4 Department of Operative Medicine, Clinic of Visceral, Transplant and Thoracic Surgery, Innsbruck Medical University, Innsbruck, Austria; New York University, United States of America

## Abstract

**Background:**

Pro-inflammatory, cytotoxic CD4^+^CD28^−^ T-cells with known defects in apoptosis have been investigated as markers of premature immuno-senescence in various immune-mediated diseases. In this study we evaluated the influence of polyclonal antilymphocyte globulins (ATG-Fresenius, ATG-F) on CD4^+^CD28^−^ T-cells *in vivo* and *in vitro*.

**Principal Findings:**

Surface and intracellular three colour fluorescence activated cell sorting analyses of peripheral blood mononuclear cells from 16 consecutive transplant recipients and short-term cell lines were performed. *In vivo*, peripheral levels of CD3^+^CD4^+^CD28^−^ T-cells decreased from 3.7±7.1% before to 0±0% six hours after ATG-F application (P = 0.043) in 5 ATG-F treated but not in 11 control patients (2.9±2.9% vs. 3.9±3.0%). *In vitro*, ATG-F induced apoptosis even in CD4^+^CD28^−^ T-cells, which was 4.3-times higher than in CD4^+^CD28^+^ T-cells. ATG-F evoked apoptosis was partially reversed by the broad-spectrum caspase inhibitor benzyloxycarbonyl (Cbz)-Val-Ala-Asp(OMe)-fluoromethylketone (zVAD-fmk) and prednisolon-21-hydrogensuccinate. ATG-F triggered CD25 expression and production of pro-inflammatory cytokines, and induced down-regulation of the type 1 chemokine receptors CXCR-3, CCR-5, CX3CR-1 and the central memory adhesion molecule CD62L predominately in CD4^+^CD28^−^ T-cells.

**Conclusion:**

In summary, *in vivo* depletion of peripheral CD3^+^CD4^+^CD28^−^ T-cells by ATG-F in transplant recipients was paralleled *in vitro* by ATG-F induced apoptosis. CD25 expression and chemokine receptor down-regulation in CD4^+^CD28^−^ T-cells only partly explain the underlying mechanism.

## Introduction

Polyclonal antilymphocyte or antithymocyte globulins (ATG) are potent immunosuppressive agents used in organ transplantation since the late 1960s. Clinical indications for ATGs include not only prevention (induction therapy) or rescue treatment of acute rejection, conditioning for haematopoietic stem cell transplantation from unrelated HLA-matched or haploidentical donors, treatment of graft-versus-host-disease [1] and severe aplastic anaemia [2], but also a rescue therapy in severe and therapy refractory rheumatic diseases [3], [4].

The precise action mechanism of ATGs is still undefined. Human thymocytes are the most common source of antigens for the preparation of ATGs, thus including a mixture of multiple antibodies to various lymphocyte surface antigens [5]–[7]. Induction of profound lymphocytopenia as well as functional immunomodulation with down-regulation of leukocyte adhesion molecules, binding of chemokine receptors and interaction with receptors of lymphocyte activation [1] have been described as potential contributors to the immunosuppressive effects of ATGs. Lymphocyte depletion is caused by complement-mediated cytolysis, clearance of lymphocytes through opsonization and phagocytosis by macrophages, induction of Fas-mediated apoptosis and Cathepsin B dependent mechanisms of activated and non-activated lymphocytes [8]–[10]. Functional antibodies of rabbit ATGs to leukocyte adhesion molecules and chemokine receptors impair responses to chemotactic signals and lymphocyte trafficking to sites of inflammation [7], [11]. Furthermore, ATGs trigger apoptosis in B-cell lineages, interfere with functional properties of dendritic cells and induce the expansion and activation of regulatory and natural killer T-cells [12]–[14].

Recently, a subgroup of pro-inflammatory T-cells has been identified in patients with various chronic inflammatory diseases [15]–[21] and allograft recipients [22], [23]. CD4^+^CD28^−^ T-cells are resistant against apoptotic stimuli and are suggested to perpetuate chronic inflammation [24], [25]. Clinically, these cells were associated with the severity of rheumatoid arthritis and rheumatoid arthritis-associated vasculitis [26]–[28], ankylosing spondylitis [18], Wegeners' granulomatosis [20], [21], contributed to plaque instability in patients with coronary artery disease [29], [30], and were linked with stroke recurrence and increased mortality of patients following ischemic stroke [31]. Besides, increased levels of CD4^+^CD28^−^ T-cells were associated with deterioration of graft function after kidney and lung transplantation [22], [32]. Functionally, these T-cells are capable of releasing large amounts of interferon-γ (IFN-γ), perforin and granzyme B, providing them with the possibility to lyse target cells [30]. Especially the cytolytic proteins perforin and granzyme B are involved in the process of allograft deterioration and may be useful in predicting graft loss [33], [34]. Overall, CD4^+^CD28^−^ T-cells are a marker for chronic inflammation, early aging [35], and compromised immunocompetence [36].

Depletion of this “nasty” pro-inflammatory cytotoxic T-cell subset seems to be a promising new therapeutic approach; however, up to now, only 33–36% decrement of peripheral CD4^+^CD28^−^ T-cells was observed after treatment with the anti-tumor necrosis factor-α (TNF-α) antibody, infliximab [37]–[40]. Besides, results from a retrospective, observational study suggest an even lower reduction of the frequency of peripheral CD4^+^CD28^−^ T-cells by statins in unstable angina [41].

The aim of this study was to evaluate the effects of ATG-Fresenius (ATG-F) on circulating pro-inflammatory CD3^+^CD4^+^CD28^−^ T-cells. Observing a complete depletion of this lymphocyte population by ATG-F treatment *in vivo*, we then investigated dose-dependent pro-apoptotic and immunomodulatory effects of ATG-F on CD4^+^CD28^−^ T-cells *in vitro*.

## Results

### Rapid depletion of circulating CD3^+^CD4^+^CD28^−^ T-cells in transplant patients treated with polyclonal antilymphocyte globulins

Demographic data of patients undergoing organ transplantation with or without ATG-F treatment are shown in Table 1.

**Table 1:** Patients' characteristics at enrolment of the study.

ATG treatment*	Sex †	Age [years]	CRP [mg/dl]♣	Leukocytes [G/l]♦	Hb [g/l]	concommitant immune-suppressive therapy‡	TX-organ	treated condition
N	M	56	0.7	9.2	158	CsA, MMF, GC, aIL2R	L	chronic emphysema
N	M	57	2.3	5.0	108	CsA, GC, MMF	K	chronic GN
N	M	46	0.7	4.6	126	aIL2R, FK-506, GC	L	CirrhHep
N	M	43	0.7	8.3	117	aIL2R, FK-506, GC	K	chronic GN
N	M	61	0.7	2.9	100	aIL2R, FK-506, GC	L	CirrhHep
N	F	50	3.5	4.8	100	aIL2R, RAP, MMF, GC	K	PN
N	M	44	0.7	4.4	162	CsA, GC, MMF	K	Alport syndrome
N	M	45	0.7	10.6	136	FK-506, GC, MMF	K	chronic graft rejection
N	F	59	0.4	9.0	129	aIL2R, FK-506, MMF,GC	K	PN
N	M	44	0.3	9.3	129	FK-506, MMF, GC	K	IgA nephropathy
N	M	51	0.3	3.8	142	FK-778, FK-506, GC	L	CirrhHep
Y	M	51	0.7	4.7	133	ATG, FK-506, RAP, GC	K, P	DM I
Y	F	48	0.7	6.6	108	ATG, FK-506, RAP, GC	K, P	DM I
Y	F	29	0.3	8.0	105	ATG, FK-506, MMF, GC	K, P	DM I
Y	F	24	0.4	7.9	112	ATG FK-506, RAP, GC	K, P	DM I
Y	M	41	0.5	7.1	155	ATG, FK-506, MMF, GC	K, P	DM II

* no, N; yes, Y;

† male, M; female, F;

♣ C-reactive protein ,CRP (normal values: 0–0.6 mg/dl);

♦ normal values: 3.8–10.5 G/l; hemoglobin, Hb (normal values: 120–157 g/l).

‡ cyclosporine A, CsA; mycophenolate mofetil, MMF; glucocorticoids, GC; monoclonal anti-human IL-2 receptor antibodies (daclizumab, zenapax™), aIL2R; tacrolimus, FK-506; rapamycine, RAP; monoclonal anti-human CD52 antibodies (alemtuzumab, campath™), anti-CD52 Ab; leflunomide analogue FKK-778, FKK-778; ATG Fresenius, ATG.

Decompensated cirrhosis hepatis, CirrhHep; diabetes mellitus with nephropathy, DM; glomerulonephritis, GN; Kidney, K; lung, L; pancreas, P; polycystic nephropathy, PC; transplant, TX.

**Figure 1:**
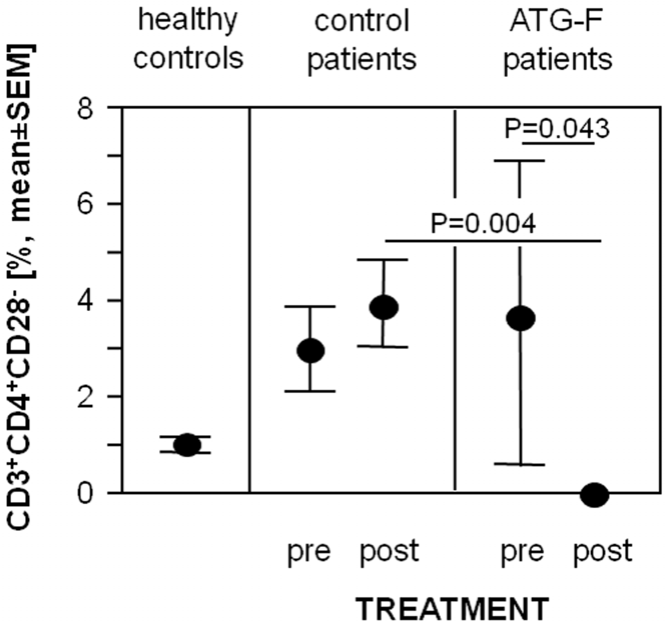
Treatment with polyclonal antilymphocyte globulins induces depletion of circulating CD3^+^CD4^+^CD28^−^ T-cells in transplant recipients. Prevalences of peripheral circulating CD3^+^CD4^+^CD28^−^ T-cells in 16 age- and sex-matched healthy controls, 5 allograft recipients before and 6 hours after the application of ATG-F and 11 control patients before and 6 hours after organ transplantation. Data are given as mean±standard error of the mean (SEM).

In this small pilot study the prevalence of peripheral circulating CD3^+^CD4^+^CD28^−^ T-cells was tested before and 6 hours after organ transplantation with and without application of ATG-F. Results of a blinded laboratory investigator showed that ATG-F treatment resulted in a total decrement of peripheral T-cells. Peripheral levels of circulating CD3^+^CD4^+^CD28^−^ T-cells decreased from 3.7±7.1% to 0±0% (P = 0.043) in ATG-F treated patients, but did not decrease in control patients (2.9±2.9% and 3.9±3.0% before and after organ transplantation, respectively, Figure 1).

### Antilymphocyte globulins preferentially trigger apoptosis in CD4^+^CD28^−^ T-cells in vitro

**Figure 2:**
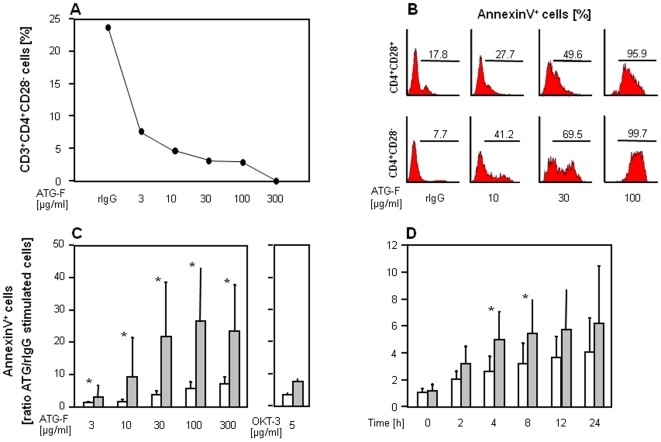
Antilymphocyte globulins preferentially trigger apoptosis in CD4^+^CD28^−^ T-cell subsets *in vitro*. *(* ***A*** *)* Influence of different doses of ATG-F on the prevalence of CD3^+^CD4^+^CD28^−^ T-cells in one representative patient. ATG-F or rabbit IgG (rIgG) were applied for 18 hours, as indicated. *(* ***B*** *)* Representative histograms showing ATG-F induced apoptosis measured by AnnexinV staining in various doses in comparison to unspecific rabbit IgG by three colour FACS analysis. Gates were set on lymphocytes (forward and sideward scatter) as well as on CD4^+^CD28^+^ and CD4^+^CD28^−^ cells (not shown), and markers according to the negative control. ATG-F induced apoptosis in both, CD28^+^ and CD28^−^CD4^+^ T-cell subsets in a dose- (n = 6) *(* ***C*** *)* and time dependent manner (30 µg/ml ATG-F, n = 6) *(* ***D*** *)* measured by AnnexinV staining in flow cytometry. Data are given as mean (bars) and standard deviation (lines) for CD4^+^CD28^+^ (white) and CD4^+^CD28^−^ T-cells (grey). To account for the different rate of apoptosis in unstimulated CD28^+^ and CD28^−^CD4^+^ T-cells, AnnexinV^+^ cells are depicted as the ratio of ATG-F versus rabbit IgG stimulated cells. An asterisk indicates significant differences (P<0.05) between CD28^+^ and CD28^−^CD4^+^ T-cell subsets. Significances between rabbit IgG and ATG-F evoked apoptosis were found at doses of 30–300 µg/ml ATG-F in CD4^+^CD28^+^ and at doses of 3–300 µg/ml ATG-F in CD4^+^CD28^−^ T-cells (each with P<0.05). In the time course the ATG-F triggered effect on apoptosis was first detectable after 2 hours in both CD4^+^ T-cell subsets (each with P<0.05).

I*n vitro*, treatment of short term cell lines with ATG-F resulted in a preferential reduction of CD3^+^CD4^+^CD28^−^ compared to CD28^+^ T-cells. Figure 2A shows the prevalences of CD3^+^CD4^+^CD28^−^ after exposure to rabbit IgG and different doses of ATG-F over 18 hours in a representative example.

ATG-F induced significant apoptosis in both, CD28^+^ and CD28^−^CD4^+^ T-cell subsets in a dose- and time-dependent manner (Figures 2B, C and D). Stimulation with 3–300 µg/ml ATG-F for 18 hours led to a mean 4.3 times higher apoptosis rate in CD28^−^ compared to CD28^+^CD4^+^ T-cells. The effect of 30 µg/ml ATG-F on apoptosis was first detected after 2 hours of stimulation and was maximal after 24 hours in both CD4^+^ T-cell subsets. Notably, depletion of CD28^−^ T-cells *in vitro* was incomplete with ATG-F at a dose of 100 µg/ml corresponding to *in vivo* blood concentrations after treatment with rabbit ATGs [8].

### Antilymphocyte globulin triggered apoptosis of CD4^+^ T-cells partially depends on activation of caspases, but not on the Fas-receptor or IL-2 pathway

A Cathepsin-B- and Fas-receptor mediated mechanism were analysed to investigate the underlying mechanisms of apoptosis induced in CD4^+^CD28^−^ T-cells by ATG-F.

**Figure 3:**
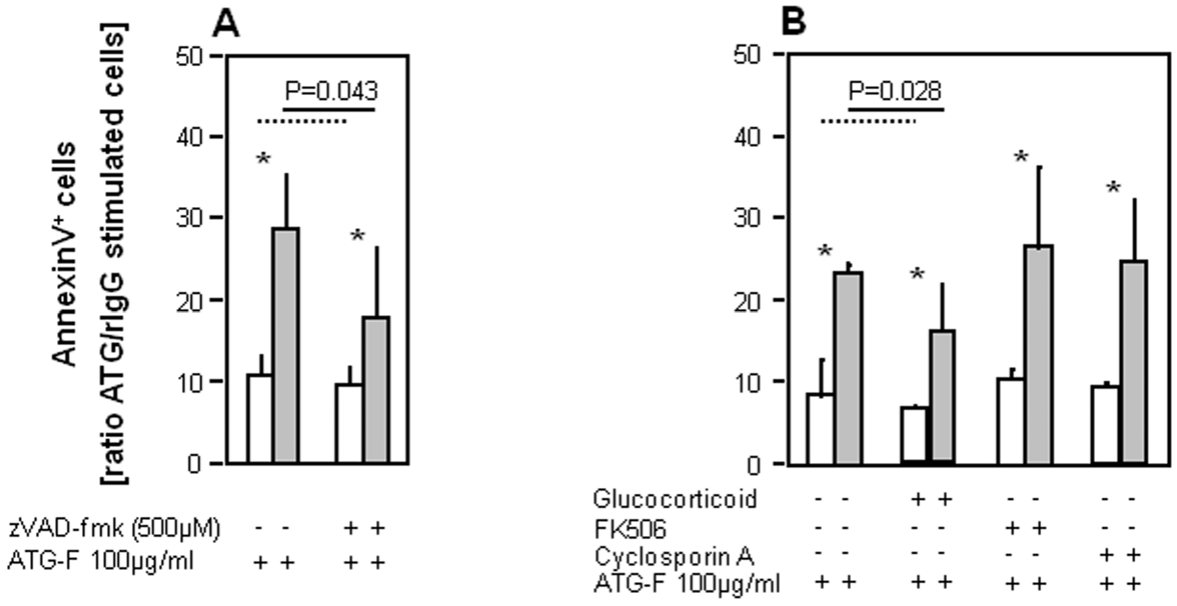
Antilymphocyte globulin-triggered apoptosis of CD4^+^ T-cells partially depends on activation of caspases. To investigate the underlying mechanisms of apoptosis induction in CD4^+^ T-cells by ATG-F *(* ***A*** *)* a caspase-mediated mechanism (n = 5) was analysed by AnnexinV staining in flow cytometry. *(* ***B*** *)* As the immunosuppressive agents prednisolon-21-hydrogensuccinate (glucocorticoid), FK506 and cyclosporine A are known to interfere with the interleukin (IL)-2 pathway, their influence on ATG-F mediated apoptosis was examined in CD28^+^ and CD28^−^CD4^+^ T-cell subsets (n = 6). Data are given as mean (bars) and standard deviation (lines) for CD4^+^CD28^+^ (white) and CD4^+^CD28^−^ T-cells (grey). An asterisk indicates significant differences (P<0.05) between CD28^+^ and CD28^−^CD4^+^ T-cell subsets. Depicting significances broken and continuous lines were used for CD28^+^ and CD28^−^CD4^+^ T-cells, respectively.

Earlier studies had suggested that ATGs induced T-cell apoptosis by a Cathepsin-B-dependent mechanism [10]. Our T-cells from short term cell lines incubated with the broad-spectrum caspase inhibitor benzyloxycarbonyl (Cbz)-Val-Ala-Asp(OMe)-fluoromethylketone (zVAD-fmk) before stimulation with ATG-F resulted in a partial reversion of ATG-F evoked apoptosis at a dosage of 100 µg/ml in both CD4^+^ T-cell subsets (each with P = 0.043; Figure 3A). This effect of zVAD-fmk on ATG-F caused apoptosis was 9.8 times greater in CD4^+^CD28^−^ compared to CD4^+^CD28^+^ T-cells (P = 0.043).

As submitogenic ATG dosages (10–100 µg/ml) induce the expression of the Fas-receptor (Apo-1, CD95) and Fas-ligand (Fas-L, CD95-L) on activated lymphocytes resulting in Fas-mediated apoptosis [9], we then studied a possible ATG-F-triggered Fas-receptor mediated pathway of apoptosis in CD28^+^ and CD28^−^CD4^+^ T-cell subsets. Short term cell lines were pre-incubated with the inhibiting anti-Fas Ab ZB4, blocking the interaction between Fas-receptor and Fas-L, before addition of the activating anti-Fas Ab CH11 (positive control) or ATG-F. The inhibiting anti-Fas Ab ZB4 reversed the effect of the activating CH11 in both, CD28^+^ and CD28^−^CD4^+^ T-cell subsets, but had no effect on ATG-F induced apoptosis in both CD4^+^ T-cell subsets at a dosage of 100 µg/ml (Figure S1), thus rendering a Fas-receptor dependent effect of ATG-F improbable.

As the next step forward we analysed the effects of prednisolon-21-hydrogensuccinate, FK506 and cyclosporine A (CsA) on ATG-F-induced cell death as these immunosuppressive agents interfere with the IL-2 pathway, as IL-2 is required for acquisition of susceptibility to Fas-mediated apoptosis regulating the transcription and surface expression of FasL on lymphocytes [42]. As shown in Figure 3B, the addition of prednisolon-21-hydrogensuccinate partially reversed ATG-F caused apoptosis in both, CD28^+^ and CD28^−^CD4^+^ T-cell subsets (each with P = 0.028) with a 4.1 greater effect in CD4^+^CD28^−^ T-cells (P = 0.028). However, FK506 and CsA had no effect on ATG-F evoked apoptosis in both CD4^+^ T-cell subsets, thus excluding a possible role of the IL-2 pathway on ATG-F induced apoptosis.

### Chemokine receptors and transendothelial migration: down-regulation of T helper 1 type chemokine receptors on CD4^+^CD28^−^ T-cells by antilymphocyte globulins

**Figure 4:**
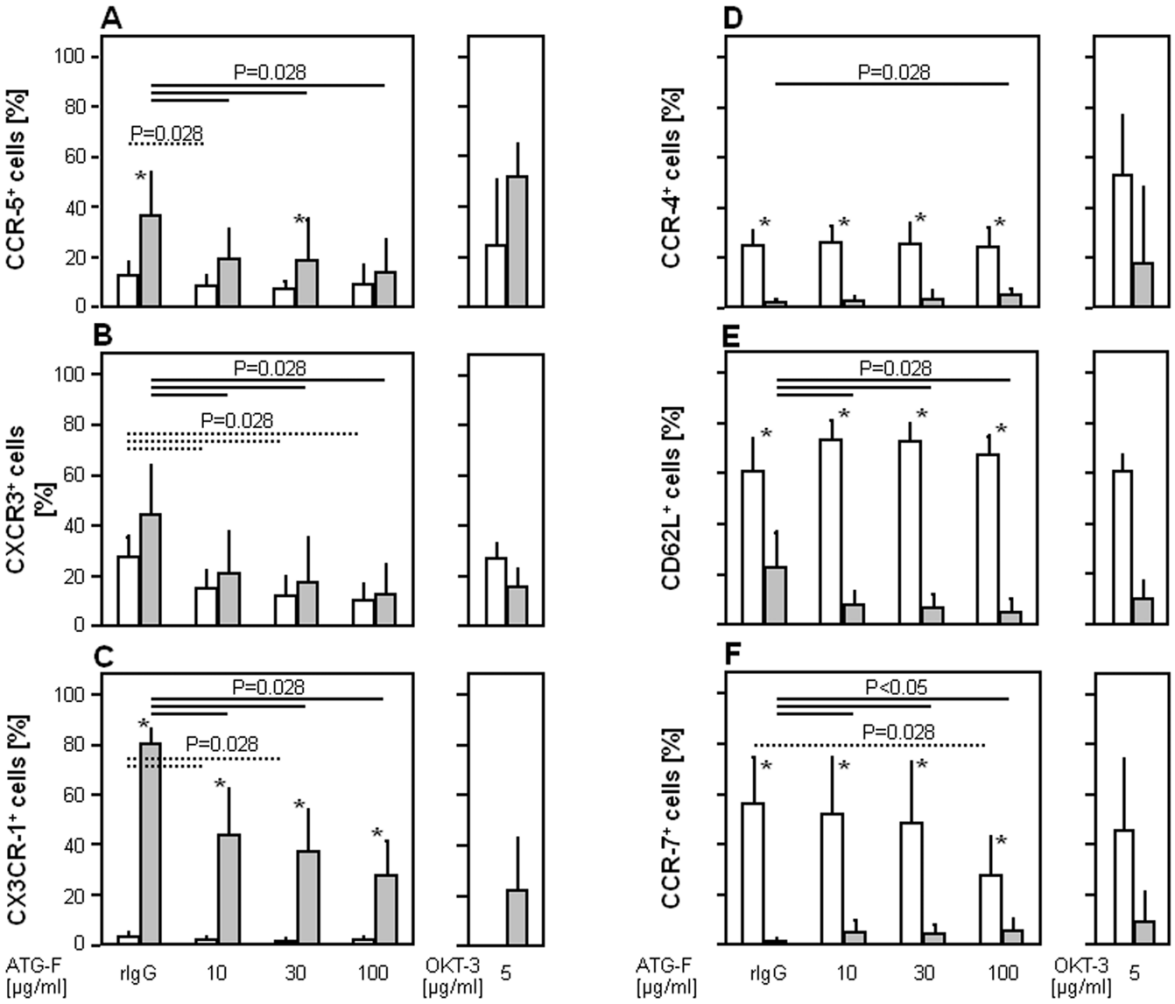
Down-regulation of Th1 type chemokine and leukocyte homing receptors by antilymphocyte globulins in CD4^+^CD28^−^ T-cells. Dose dependent *in vitro* effects of ATG-F on surface expression of the type 1 chemokine receptors *(* ***A*** *)* CCR-5, *(* ***B*** *)* CXCR-3, *(* ***C*** *)* CX3CR-1, the type 2 chemokine receptor *(* ***D*** *)* CCR-4, and the central memory receptors *(* ***E*** *)* CD62L and *(* ***F*** *)* CCR-7 on CD4^+^ T-cell subsets in comparison to rabbit IgG (rIgG) (n = 6). Data are given as mean (bars) and standard deviation (lines) for CD4^+^CD28^+^ (white) and CD4^+^CD28^−^ T-cells (grey). An asterisk indicates significant differences (P<0.05) between CD28^+^ and CD28^−^CD4^+^ T-cell subsets. Depicting significances between rabbit IgG and ATG-F induced modulation of chemokine receptor expression broken and continuous lines were used for CD28^+^ and CD28^−^CD4^+^ T-cells, respectively.

The expression of chemokine receptors and leukocyte trafficking were studied as possible targets of ATG-F effects. Stimulation of peripheral blood mononuclear cells (PBMCs) with ATG-F at a dosage of 100 µg/ml for 4 hours resulted in a preferential down-regulation of T helper (Th) 1 type chemokine receptors CCR-5 (mean difference between ATG-F and rabbit IgG stimulated cells: −20.7% out of CD28^−^ vs. −3.6% out of CD28^+^CD4^+^ T-cells, P = 0.028, Figure 4A), CXCR-3 (−31.7% vs. −17.3%, P = 0.028, Figure 4B) and CX3CR-1 (−51.9% vs. −1.3%, P = 0.028, Figure 4C) as well as the central memory adhesion molecule CD62L (−17.7% vs. −6.5%, P = 0.028, Figure 4E) on CD4^+^CD28^−^ T-cells compared to CD28^+^ T-cells. The surface expression of Th2 type CCR-4 slightly increased on CD4^+^CD28^−^ (+2.8% positive cells, P = 0.028, Figure 4D) but not on CD28^+^ T-cells and the surface expression of the central memory chemokine receptor CCR-7 was differentially influenced by ATG-F with an up-regulation on CD4^+^CD28^−^ (+4.5%, P = 0.028) and a down-regulation on CD4^+^CD28^+^ T-cells (−26.3%, P = 0.028, Figure 4F).

The transendothelial migration assay was performed to investigate the functional consequences of ATG-F mediated changes of chemokine receptor expression. Based on the results of CX3CR-1 reported above we used the recombinant human chemokine fractalkine (sFKN, CX3CL-1) as a ligand. The chemokine receptor CX3CR-1 was almost exclusively expressed on CD4^+^CD28^−^ T-cells and strongly influenced by ATG-F treatment. Transendothelial migration evoked by sFKN was partially reversed by the pre-incubation of CD4^+^ isolated T-cells with 100 µg/ml ATG-F for 2 hours (expressed as transmigration index with 1.4±0.3 vs. 1.0±0.6; P = 0.033; data not shown).

### Cytokine production mediated by supramitogenic dosages of antilymphocyte globulins in CD4^+^CD28^−^ T-cells

General symptoms induced by a massive cytokine release *in vivo* occur even hours after ATG administration. To investigate the possible contribution of CD28^−^ T-cells to these adverse reactions at the lymphocyte level, cytokine production of CD4^+^CD28^−^ was tested upon stimulation with ATG-F *in vitro*.

**Figure 5:**
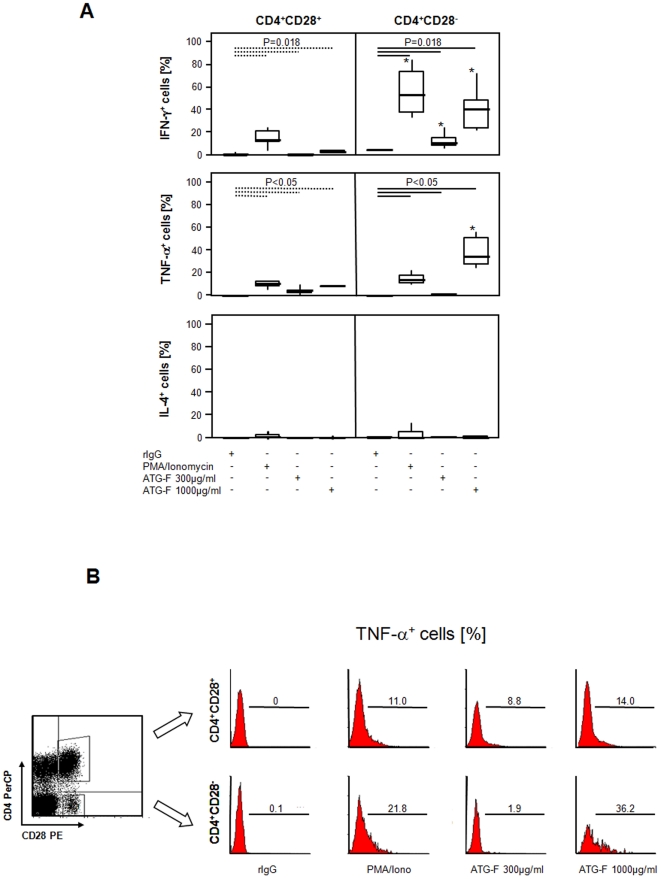
Production of pro-inflammatory cytokines by polyclonal antilymphocyte globulins in CD4^+^CD28^−^ T-cells. *(* ***A*** *)* To test whether ATG-F treatment leads to cytokine production of CD4^+^ T-cell subsets *in vitro*, stimulation with rabbit IgG (rIgG), phorbol 12-myristate 13-acetate (PMA)/ionomycin, and ATG-F at a dose of 300 µg/ml or 1000 µg/ml and intracellular cytokine staining for IFN-γ, TNF-α and IL-4 was performed (n = 6). Whiskers box plots show 50% of cases within the boxes and all data excluding mavericks between the end-points of the whiskers (lines). An asterisk indicates significant differences (P<0.05) between CD28^+^ and CD28^−^CD4^+^ T-cell subsets. Depicting significances between rabbit IgG, PMA/ionomycin and ATG-F induced cytokine expression broken and continuous lines were used for CD28^+^ and CD28^−^CD4^+^ T-cells, respectively. *(* ***B*** *)* Representative dot plot and histograms depicting ATG-F induced production of TNF-α after 4 hours of stimulation in supramitogenic dosages of 300 µg/ml and 1000 µg/ml in comparison to stimulation with unspecific rIgG and PMA/ionomycin in the presence of brefeldin A. Gates were set on lymphocytes (forward and sideward scatter) as well as on CD4^+^CD28^+^ and CD4^+^CD28^−^ cells, and markers according to the negative control.

Baseline levels of intracellular cytokines were negligible in both CD4^+^ subsets. Incubation of cells with ATG-F at dosages of 3–300 µg/ml led to the activation of both, CD28^−^ and CD28^+^CD4^+^ T-cell subsets as indicated by increased CD25 expression (Figure S2). Application of ATG-F at dosages of 300 µg/ml and 1000 µg/ml for 4 hours stimulated the production of IFN-γ and TNF-α but not that of IL-4 in both CD4^+^ T-cell subsets. 1000 µg/ml ATG-F resulted in a 6.2 times and 4.6 times higher effect on IFN-γ and TNF-α production, respectively, in CD28^−^ T-cells compared to their CD28^+^CD4^+^ T-cell counterparts (Figures 5A and B).

## Discussion

Recent studies indicate that pro-inflammatory CD28^−^ T-cells persist over years and include most of the oligoclonally expanded T-cells. The clonal outgrowth of these CD4^+^CD28^−^ T-cells is associated with defects in apoptotic pathways caused by inadequate upregulation of bcl-2 [24] and Fas-associated death domain-like IL-1-converting enzyme-like inhibitory protein, an inhibitor of Fas signalling that is normally degraded in T-cells following activation in the presence of IL-2 [25]. Clinically, these specific T-cells are responsible for the chronicity and relapsing nature of immune-mediated rheumatic diseases. Thus, depletion of pro-inflammatory, cytotoxic CD3^+^CD4^+^CD28^−^ T-cells by ATG-F treatment *in vivo* provides a promising new therapeutic concept.

For this effect we demonstrated increased *in vitro* apoptosis of CD4^+^CD28^−^ T-cells compared to CD28^+^ T-cells that was partially regulated by caspases and a yet undefined IL-2 independent mechanism inhibited by prednisolon-21-hydrogensuccinate:

1. Previous reports had shown that supramitogenic doses of ATGs led to apoptosis of peripheral blood lymphocytes from healthy donors and myeloma cells depending on caspases, particularly on Cathepsin B [10], [43]. Indeed, caspase-mediated apoptotic pathways are still functional in CD4^+^CD28^−^ T-cells, as the broad spectrum caspase inhibitor zVAD-fmk significantly reduced ATG-F induced cell death.
2. Concerning ATG-induced apoptosis of pre-activated lymphocytes by either Fas-receptor/FasL-dependent pathways [9], [10], ATG-F evoked apoptosis of CD4^+^CD28^−^ T-cells, but was not influenced by the neutralizing anti-Fas-antibody ZB4, suggesting that the described defects in the Fas-receptor/FasL pathway of CD4^+^CD28^−^ T-cells cannot be restored by ATG-F treatment [25].
3. The role of IL-2 for ATG-F induced apoptosis of CD4^+^CD28^−^ T-cells remains unclear. As IL-2 signalling has been shown to enhance susceptibility to Fas-receptor/FasL mediated apoptosis, we expected IL-2 inhibition to decrease the apoptosis-rate of CD4^+^CD28^+^ T-cells, but not to affect CD4^+^CD28^−^ T-cells [9], [42]. Surprisingly, the IL-2 inhibitors FK506 and CsA had no effect on ATG-F evoked apoptosis in any CD4^+^ T-cell subpopulations, whereas the addition of prednisolon-21-hydrogensuccinate resulted in a partial reduction of apoptosis in both CD28^+^ and CD28^−^CD4^+^ T-cell subsets. It can only be speculated that prednisolon-21-hydrogensuccinate influences apoptosis of CD4^+^ T-cells by IL-2 independent mechanisms and that the divergences between our and previous results concerning FK506 and CsA are caused by differences in the experimental settings [9]: Our analyses were performed with a different ATG preparation and shorter pre-incubation times. Besides, we used T-cells from short term cells lines instead of freshly isolated lymphocytes activated with phytohemagglutinin [9].

Apart from their pro-apoptotic effects on lymphocytes, ATGs are known to be immunomodulatory influencing leukocyte chemotaxis and trafficking [11], [12]. As apoptosis of CD4^+^CD28^−^ T-cells was incomplete after incubation with submitogenic doses of ATG-F in our *in vitro* studies but *in vivo* treatment resulted in depletion of CD3^+^CD4^+^CD28^−^ T-cells, we tested whether ATG-F forces increased homing of this pro-inflammatory T-cell subpopulation. Indeed CD4^+^CD28^−^ T-cells are unique to combine receptors of short-lived memory effector cells and long-lived central memory cells enabling these cells to home to both, peripheral inflammatory lesions as well as organized lymphoid organs [44]. Interestingly, our experiments showed a down-regulation of the Th1 type and effector memory receptors CCR-5, CXCR-3 and CX3CR-1 as well as the central memory adhesion molecule CD62L on CD4^+^CD28^−^ T-cells after stimulation with ATG-F *in vitro*. Besides, ATG-F reduced migration of CD4^+^CD28^−^ T-cells in a transendothelial assay confirming the functional relevance of changed chemokine and chemokine receptor expression. Taken together, these data suggest that depletion of CD4^+^CD28^−^ T-cells *in vivo* is not caused by increased cell trafficking and that other, yet undefined mechanisms may contribute to complete disappearance of peripheral circulating CD4^+^CD28^−^ T-cells.

In the patients treatment of ATG-F is concerned to induce lymphocytic cytokine release causing the “first dose syndrome”. Our *in vitro* cytokine data for the CD4^+^CD28^+^ T-cells are in line with previous studies revealing an ATG-F promoted production of IFN-γ and TNF-α with a shift toward a Th1 cytokine profile [45], [46]. The effect of ATG-F on CD4^+^CD28^−^ T-cells, however, was much stronger leading to a concept that high pre-treatment levels of circulating CD4^+^CD28^−^ T-cells could be a risk factor for infusion related adverse reactions after high dosages of ATG-F. Because of the small sample size of our cohort we were unable to test this hypothesis in this study.

The small and heterogenous group of enrolled transplant recipients in our *in vivo* study and the short follow-up period of patients at one single time point after ATG-F application is certainly the main limitation of this study. Thus, a type 1 error with overestimation of the ATG-F induced effect of the presented *in vivo* data cannot be excluded. Phenotypical and functional differences between CD4^+^CD28^−^ T-cells from patients ≥50 years and those from younger people were previously suggested; however, we observed no influence of age on ATG-F mediated T-cell apoptosis in our *in vitro* and *in vivo* experiments. Another limitation of our study is the use of one ATG product, namely ATG-F, for our *in vitro* and *in vivo* analyses, although different ATG preparations are currently available. As ATGs contain a mixture of multiple antibodies to various lymphocyte surface antigens, we cannot exclude that other types of ATG may have different effects on CD4^+^CD28^−^ T-cells [9], [47], [48]. Our data on ATG-F evoked apoptosis measured by AnnexinV FACS analysis show a certain variability possibly related to the examinations of samples from different patients and the individually different susceptibility to ATG-F. Besides, AnnexinV staining was the only (although well established) method to determine apoptosis. Apoptosis induction, however, was reversible thus excluding unspecific effects and the conclusions drawn from different experiments were the same: CD4^+^CD28^−^ T-cells, otherwise known as resistant to apoptosis are more susceptible to ATG-F evoked apoptosis than their CD28^+^ counterparts. Concerning the assays to explore the *in vitro* mechanisms, we identified 2 potential pathways triggering apoptosis of CD4^+^CD28^−^ T-cells after ATG-F stimulation, but additional mechanisms restoring the capacity of CD4^+^CD28^−^ T-cell to undergo apoptosis most likely exist as the addition of the broad spectrum caspase inhibitor zVAD-fmk and prednisolon-21-hydrogensuccinate resulted only in partial reversions of ATG-F evoked apoptosis. Thus, we could not explain why CD4^+^CD28^−^ T-cells totally disappear from peripheral blood after ATG-F therapy despite incomplete *in vitro* apoptosis and downregulation of homing and effector chemokine receptors. One untested, but possible explanation is an upregulation of CD28 on surviving (non-apoptotic) CD4^+^CD28^−^ T-cells upon ATG-F treatment, however, future research is needed to clarify this issue. We concede, that the long-term consequences of ATG-F-induced depletion of circulating peripheral and tissue infiltrating CD4^+^CD28^−^ T-cells in chronic inflammatory diseases is unclear and may result in a driving force to re-fill-up peripheral niches achieving T-cell homoeostasis. Also this issue has to be addressed by a longitudinal follow-up study.

In summary, ATG-F is the first substance effectively depleting circulating CD3^+^CD4^+^CD28^−^ T-cells in patients undergoing organ transplantation at least over 6 hours. ATG-F preferentially induces apoptosis of this CD4^+^CD28^−^ T-cell subset *in vitro* and this effect is partially reversed by the broad-spectrum caspase inhibitor zVAD-fmk and prednisolon-21-hydrogensuccinate. Additionally, stimulation with ATG-F results in a predominant Th1-type activation with IFN-γ and TNF-α cytokine production, as well as functional down-regulation of effector memory and central memory chemokine receptors of CD4^+^CD28^−^ T-cells.

## Methods

### Statement of the ethics committee

The ethics committee of the Innsbruck Medical University granted the study during their session number 225/4.19, study number UN2239 on the 01.02.2005.

### Patients' recruitment and antilymphocyte globulins

Sixteen consecutive recipients of either kidney or lung transplant were enrolled into the study (Table 1, patients' characteristics). Blood samples were drawn from five patients (38.6±11.8 years old, 3 female patients) receiving ATG-F (Fresenius Biotech, Gräfelfing, Germany) preoperatively in a dosage of 8 mg/kg body weight and from 11 patients without ATG-F treatment (50.5±6.7 years old, 2 female patients). Percentages of CD28^−^ out of the CD3^+^CD4^+^ PBMCs were determined before and 6 hours after ATG-F application by flow cytometry. Laboratory investigators were blinded for ATG-F treatment of patients.

For *in vitro* analyses consecutive patients suffering from chronic inflammatory diseases with a prevalence of peripheral CD3^+^CD4^+^CD28^−^ T-cells ≥5% were included into the study. Patients with immune-mediated diseases were selected, as functional differences between CD28^−^ T-cells from different diseases or in the elderly have not been detected in earlier studies. Blood samples were drawn after informed and written consent as approved by the ethics committee of the Innsbruck Medical University, Austria (AN 2239 - 225/4.19, PN 10/2005).

Within the context of this report, ATG is used to refer to either antithymocyte or antilymphocyte globulins in general, whereas ATG-F is used to refer to antilymphocyte globulins offered by Fresenius Biotech.

### Cell preparation and short term cell lines

Peripheral venous blood was drawn and PBMCs were isolated by Ficoll density gradient centrifugation. Short-term cell lines were established from fresh PBMCs stimulated with immobilized anti-CD3 mAb (OKT3; eBioscience, San Diego, CA, USA) for 18 hours. Cells were then maintained in logarithmic growth with densities between 0.5 and 2×10^6^ cells/ml in RPMI 1640 containing 10% foetal calf serum, 2 mmol/l L-glutamine, 50 U/ml penicillin, 5 µg/ml streptomycin (all from PAA Laboratories, Linz, Austria) and 20 U/ml recombinant human IL-2 (Preprotech, London, UK). Experiments were performed later than 7 days after initiation of the culture.

### Stimulation and blocking experiments

For comparison of the dose-dependent effect of ATG-F on apoptosis as well as expression of the activation marker IL-2Rα (CD25) on CD28^+^ and CD28^−^CD4^+^ T-cell subsets, CD3^+^ T-cells from short term cell lines were incubated with submitogenic (3, 10, 30 µg/ml), mitogenic (100 µg/ml) and supramitogenic dosages (300 µg/ml) of ATG-F (Batch# RU10L-2), 5 µg/ml unspecific rabbit IgG (Fresenius) as a negative control or 5 µg/ml OKT-3 as a positive control for 18 hours. The used ATG-F concentrations correspond with the expected serum concentrations of rabbit ATG at 80 to 200 µg/ml [8], [9]. For evaluation of a caspase-dependent mechanism of ATG-F induced apoptosis in CD4^+^ T-cell subsets, cells from short term cell lines were pre-incubated with 500 µM of the broad-spectrum caspase inhibitor benzyloxycarbonyl (Cbz)-Val-Ala-Asp(OMe)-fluoromethylketone (zVAD-fmk; Bachem, Weil am Rhein, Germany, gift from Prof. Bernhard D, PhD) for 2 hours before stimulation with ATG-F (n = 5). To test a Fas-receptor mediated pro-apoptotic effect of ATG-F on CD4^+^ T-cell subsets, cells from short term cell lines were pre-incubated with 2 µg/ml (n = 6) of the Fas-blocking antibody ZB4 (IgG1, Biomed Immunotech Vienna, Austria) for at least 2 hours before addition of ATG-F, 5 µg/ml of the Fas-activating antibody CH11 (IgM, Immunotech, gift from PD Eller K, MD) as a positive control and 5 µg/ml unspecific rabbit IgG (Fresenius) as a negative control. To analyse whether IL-2 interfering pathways influence ATG-F induced apoptosis, cells from short term cell lines were treated with 1 µg/ml prednisolon-21-hydrogensuccinate, 200 ng/ml FK506 or 1 µg/ml CsA for 3 hours before addition of either 100 µg/ml ATG-F or 5 µg/ml unspecific rabbit IgG (Fresenius) (n = 6). Cells were then stimulated for 18 hours with 100 µg/ml ATG-F and apoptosis was evaluated by AnnexinV binding using flow cytometry.

For analysis of the time course of ATG-F on apoptosis of CD4^+^ T-cell subsets, CD3^+^ T-cells from short term cell lines were incubated with 30 µg/ml ATG-F at various time points (2, 4, 8, 12 and 24 hours).

To evaluate the influence of ATG-F on chemokine receptor expression, PBMCs were treated with ATG-F at various dosages (10, 30, 100 µg/ml) for 4 hours, and chemokine receptor expression was then evaluated by flow cytometry.

### Three colour surface staining, intracellular staining for cytokine production and flow cytometry

Surface staining of Ficoll density gradient purified PBMCs was performed using fluorescein isothiocyanate (FITC)-conjugated anti-AnnexinV, CD25, CD4, CCR-5, CX3CR-1, CD62L; phycoerythrin (PE)-conjugated anti-CD28, CXCR-3, CCR-4, CCR-7; and peridinin chlorophyll protein-conjugated (PerCP) anti-CD4 and CD3 mAb (all from Becton Dickinson, San Diego, CA, USA; except for anti-CCR-5, CX3CR-1 antibodies purchased from MBL International, Woburn, MA, USA).

For intracellular staining, cells were stimulated either with 5 µg/ml unspecific rabbit IgG (Fresenius), 25 ng/ml phorbol 12-myristate 13-acetate (PMA) and 1 µg/ml ionomycin (Sigma, Munich, Germany) or 300 µg/ml and 1000 µg/ml ATG-F in the presence of 10 µg/ml brefeldin A for 4 hours (Sigma). The supramitogenic dosages of ATG-F with 300 µg/ml and 1000 µg/ml were chosen, because induction of cytokine production are dose-dependent and doses as high as 1000 µg/ml stimulated TNF-α production in a previous study using horse ATG [49]. After cell surface staining for CD28 and CD4 with subsequent fixation and permeabilisation, cells were stained with FITC-conjugated anti-IFN-γ, TNF-α and IL-4 mAb or control Ig (R&D Systems, Inc., MN, USA). After fixation with 1% cell fix (Becton Dickinson) cells were analysed on a FACS-Calibur flow cytometer (Becton Dickinson). Data were analysed using WinMDI software (Version 2.8, Joseph Trotter, Scripps Research Institute, La Jolla, CA, USA).

### Transendothelial migration

For transmigration assays CD4^+^ T-cells were isolated from short term cell lines using magnetic bead labelled anti-CD4 mAb as well as Midi-MACS™ and Mini-MACS™ columns according to the manufacturer's instructions (Miltenyi Biotech, Amsterdam, The Netherlands). Purity of the isolated CD4^+^ T-cell fraction was more than 90% as determined by flow cytometry.

Human umbilical vein endothelial cells (HUVEC) from fresh placenta cords were isolated and grown to confluence at 37°C (humidified atmosphere). HUVECs of passages 1 and 2 were used for the transendothelial migration assays.

Six mm polyester filter inserts with 3 µM pores (Transwell™ Clear 3472, Costar, Cambridge, MA) were prepared by overnight incubation in 10% foetal calf serum containing medium prior to seeding cells. 3.3×10^4^ HUVECs were seeded per insert. HUVECs formed a tight permeability barrier within 3–5 days. Medium was changed three times a week.

Confluent HUVEC monolayers grown on Transwell™ growth supports were washed twice with phosphate-buffered saline (PBS). 5 ng/ml soluble recombinant human CX3CL-1 (sFKN, R&D Systems) or medium alone (control) were added to the lower compartment. Thereafter 0.1 ml of MACS™-sorted CD4^+^ T-cell suspension (2.0×10^6^ cells/ml) - untreated or pre-incubated with 100 µg/ml ATG-F for 2 hours - were added to the upper compartments and lymphocytes were allowed to migrate across the endothelial monolayers for 4 hours.

Transmigration of lymphocytes was quantified as described previously with some modification [50]. The number of migrated cells was determined by measuring the fluorescence of 2′,7′-bis-(2-carboxyethyl)-5,6-carboxyfluorescein-acetoxymethylester loaded cells using a microtitre plate reader (Cytofluor™, PerSeptive Biosystems, USA). Fluorescence was read at 530±25 nm. Each independent experiment was performed in duplicate. Data are expressed as transmigration index (TI) representing the ratio of the number of migrated lymphocytes across the endothelial monolayers in the presence or absence of the stimulating chemokine.

### Statistical analysis

Results were expressed as mean and standard deviation (SD). The Mann-Whitney test was used to compare between independent groups, and the Wilcoxon ranking test to compare between paired data from the CD28^+^ and the CD28^−^ T-cell compartments and the prevalence of CD3^+^CD4^+^CD28^−^ T-cells before and after treatment with ATG-F as appropriate. The TI of ATG-F treated and non-treated lymphocytes was compared by the paired Student's T-test. All statistical analyses were performed using the SPSS program, version 16.0 (Chicago, IL, USA). P<0.05 was considered to be statistically significant.

## Supporting Information

Figure S1 **Antilymphocyte globulin-triggered apoptosis of CD4^+^ T-cells is Fas-receptor independent.** To test whether the underlying pro-apoptotic effect of ATG-F on CD4^+^ T-cell subsets was Fas-receptor mediated, cells from short term cell lines (n = 6) were pre-incubated with 2 µg/ml of the Fas-blocking antibody ZB4 for at least 2 hours before addition of ATG-F, 5 µg/ml of the Fas-activating antibody CH11 as a positive control and 5 µg/ml unspecific rabbit IgG as a negative control. Apoptosis was evaluated after 18 hours of incubation by AnnexinV binding using flow cytometry. Data are given as mean (bars) and standard deviation (lines) for CD4^+^CD28^+^ (white) and CD4^+^CD28^−^ T-cells (grey). An asterisk indicates significant differences (P<0.05) between CD28^+^ and CD28^−^CD4^+^ T-cell subsets. Depicting significances broken and continuous lines were used for CD28^+^ and CD28^−^CD4^+^ T-cells, respectively.
(TIFF)

Figure S2 **Activation by polyclonal antilymphocyte globulins in CD4^+^CD28^−^ T-cells.** As ATG-F has known mitogenic properties, the expression of the lymphocytic activation marker CD25 was studied by three colour FACS analysis (n = 6). Data are given as mean (bars) and standard deviation (lines) for CD4^+^CD28^+^ (white) and CD4^+^CD28^−^ T-cells (grey). To account for the different activation rate in unstimulated CD28^+^ and CD28^−^CD4^+^ T-cells, CD25^+^ cells are depicted as the ratio of ATG-F versus rabbit IgG (rIgG) stimulated cells. An asterisk indicates significant differences (P<0.05) between CD28^+^ and CD28^−^CD4^+^ T-cell subsets. Significances between rabbit IgG and ATG-F triggered expression of CD25 were found at doses of 30–300 µg/ml ATG-F in both CD4^+^ T-cell subsets (each with P<0.05).
(TIFF)
