# Supplementary figures and images for: Apoptotic Effects of Antilymphocyte Globulins on Human Pro-inflammatory CD4+CD28− T-cells
Source: PLoS One. 2012 Mar 30;7(3):e33939. doi: 10.1371/journal.pone.0033939 (PMC3316508; doi:10.1371/journal.pone.0033939)

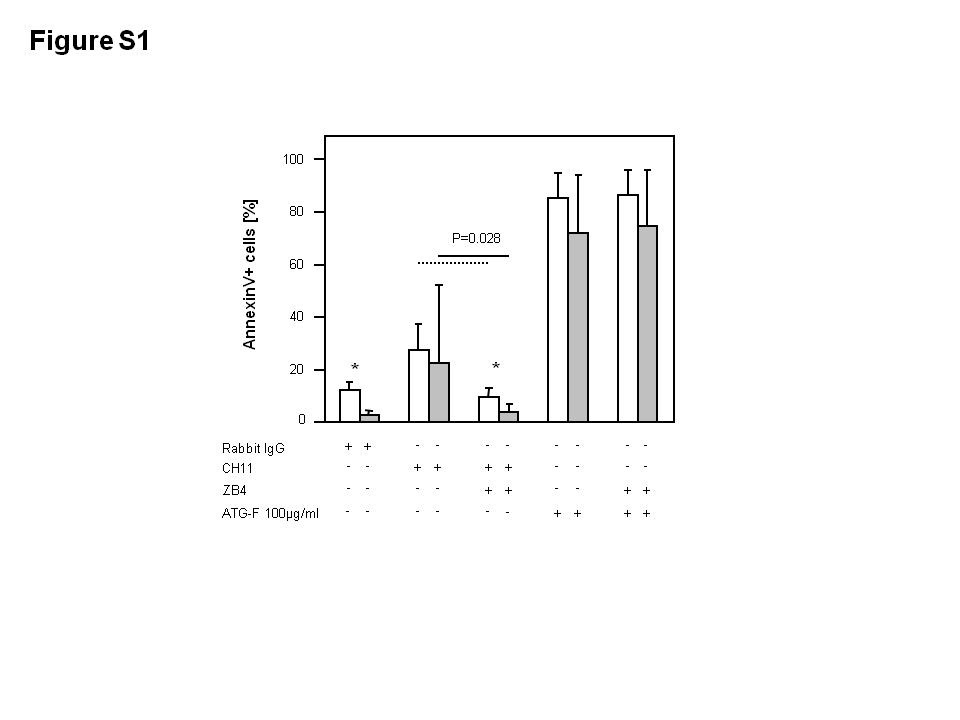

Supplement: Figure S1 — Antilymphocyte globulin-triggered apoptosis of CD4+ T-cells is Fas-receptor independent. To test whether the underlying pro-apoptotic effect of ATG-F on CD4+ T-cell subsets was Fas-receptor mediated, cells from short term cell lines (n = 6) were pre-incubated with 2 µg/ml of the Fas-blocking antibody ZB4 for at least 2 hours before addition of ATG-F, 5 µg/ml of the Fas-activating antibody CH11 as a positive control and 5 µg/ml unspecific rabbit IgG as a negative control. Apoptosis was evaluated after 18 hours of incubation by AnnexinV binding using flow cytometry. Data are given as mean (bars) and standard deviation (lines) for CD4+CD28+ (white) and CD4+CD28− T-cells (grey). An asterisk indicates significant differences (P<0.05) between CD28+ and CD28−CD4+ T-cell subsets. Depicting significances broken and continuous lines were used for CD28+ and CD28−CD4+ T-cells, respectively. (TIFF) [file pone.0033939.s001.tiff]

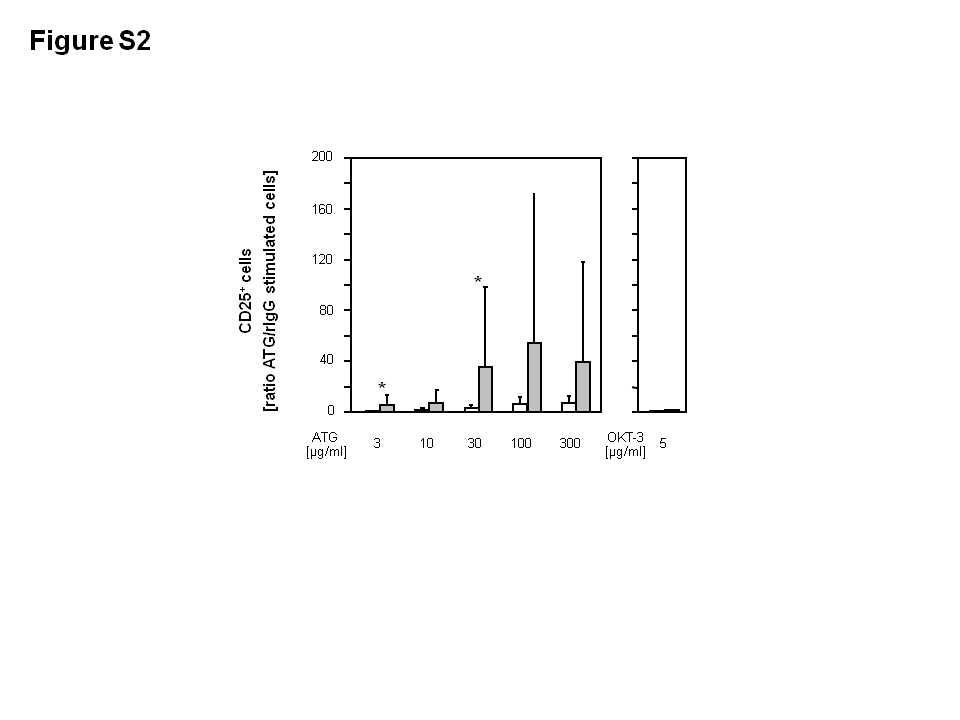

Supplement: Figure S2 — Activation by polyclonal antilymphocyte globulins in CD4+CD28− T-cells. As ATG-F has known mitogenic properties, the expression of the lymphocytic activation marker CD25 was studied by three colour FACS analysis (n = 6). Data are given as mean (bars) and standard deviation (lines) for CD4+CD28+ (white) and CD4+CD28− T-cells (grey). To account for the different activation rate in unstimulated CD28+ and CD28−CD4+ T-cells, CD25+ cells are depicted as the ratio of ATG-F versus rabbit IgG (rIgG) stimulated cells. An asterisk indicates significant differences (P<0.05) between CD28+ and CD28−CD4+ T-cell subsets. Significances between rabbit IgG and ATG-F triggered expression of CD25 were found at doses of 30–300 µg/ml ATG-F in both CD4+ T-cell subsets (each with P<0.05). (TIFF) [file pone.0033939.s002.tiff]
